# Delayed emergency healthcare seeking behaviour by Dutch emergency department visitors during the first COVID-19 wave: a mixed methods retrospective observational study

**DOI:** 10.1186/s12873-021-00449-9

**Published:** 2021-05-01

**Authors:** Maaike Nab, Robyn van Vehmendahl, Inne Somers, Yvonne Schoon, Gijs Hesselink

**Affiliations:** 1 Department of Emergency Medicine, Radboud Institute for Health Sciences, Radboud University Medical Center, Nijmegen, The Netherlands; 2 Department of Geriatrics, Radboud Institute for Health Sciences, Radboud University Medical Center, Nijmegen, The Netherlands; 3 Scientific Center for Quality of Healthcare (IQ healthcare), Radboud Institute for Health Sciences, Radboud University Medical Center, Nijmegen, The Netherlands

**Keywords:** Emergency department, COVID-19 pandemic, Delayed care

## Abstract

**Background:**

Emergency department (ED) visits due to non-coronavirus disease 2019 (COVID-19) conditions have drastically decreased since the outbreak of the COVID-19 pandemic. This study aimed to identify the magnitude, characteristics and underlying motivations of ED visitors with delayed healthcare seeking behaviour during the first wave of the pandemic.

**Methods:**

Between March 9 and July 92,020, adults visiting the ED of an academic hospital in the East of the Netherlands received an online questionnaire to collect self-reported data on delay in seeking emergency care and subsequent motivations for this delay. Telephone interviews were held with a subsample of respondents to better understand the motivations for delay as described in the questionnaire. Quantitative data were analysed using descriptive statistics. Qualitative data were thematically analysed.

**Results:**

One thousand three hundred thirty-eight questionnaires were returned (34.0% response). One in five respondents reported a delay in seeking emergency care. Almost half of these respondents (*n* = 126; 45.4%) reported that the pandemic influenced the delay. Respondents reporting delay were mainly older adults (mean 61.6; ±13.1 years), referred to the ED by the general practitioner (GP; 35.1%) or a medical specialist (34.7%), visiting the ED with cardiac problems (39.7%). The estimated median time of delay in receiving ED care was 3 days (inter quartile range  8 days). Respectively 46 (16.5%) and 26 (9.4%) respondents reported that their complaints would be either less severe or preventable if they had sought for emergency care earlier. Delayed care seeking behaviour was frequently motivated by: fear of contamination, not wanting to burden professionals, perceiving own complaints less urgent relative to COVID-19 patients, limited access to services, and by stay home instructions from referring professionals.

**Conclusions:**

A relatively large proportion of ED visitors reported delay in seeking emergency care during the first wave. Delay was often driven by misperceptions of the accessibility of services and the legitimacy for seeking emergency care. Public messaging and close collaboration between the ED and referring professionals could help reduce delayed care for acute needs during future COVID-19 infection waves.

**Supplementary Information:**

The online version contains supplementary material available at 10.1186/s12873-021-00449-9.

## Background

On March 11, 2020, the World Health Organization (WHO) declared coronavirus disease 2019 (COVID-19) a global pandemic. By the end of 2020, over 83 million COVID-19 cases have been confirmed worldwide [1]. The outbreak of COVID-19 has put unprecedented pressure on emergency healthcare worldwide. To prepare for the expected increase in healthcare demand for COVID-19 patients, hospitals repurposed inpatient beds to expand their intensive care unit (ICU) capacity and downscaled regular inpatient care. The continued spread of the virus forced many countries into a lockdown and to take active measures to prevent further COVID-19 transmission.

As COVID-19 made its way across the globe, another worrying trend evolved, namely the rapid decline of emergency admissions since the start of the pandemic for non-COVID-19 related health issues such as chest pain, acute coronary syndrome (ACS) or appendicitis [2–7]. Although this decline can be partially explained by the downscaling of regular care and a less active society, case reports and observational studies show that patients are less likely to seek emergency healthcare in fear of contracting COVID-19 [8, 9]. However, medical care delay or avoidance might exacerbate health problems and increase mortality risk associated with treatable and preventable health conditions [9, 10].

Recent months have shown the increased risk for new infection waves when governments attenuate public measures aimed at controlling the spread of the virus. Until the presence of a widespread vaccine for COVID-19, patients might continue to postpone their help request for acute health complaints. Anticipated future waves of the COVID-19 pandemic are thus likely to negatively affect a larger group of patients apart from those having COVID-19 [11–13]. So far, insight into the proportion of patients with a delayed ED presentation in the course of the first wave are limited by the population characteristics (i.e., specific health conditions and predominantly United States citizens), small sample sizes and relatively short data collection periods used in previous studies [9, 10, 14]. Moreover, insight into patients’ underlying motivations for medical care avoidance and delay have been largely unexplored [9], while this could help health services to anticipate and prevent this phenomenon during future contamination waves. Therefore, the aim of this study was to identify the magnitude, characteristics and underlying motivations of ED visitors with delayed healthcare seeking behaviour during the first COVID-19 wave in the Netherlands. Delayed healthcare seeking behaviour was defined as the patient’s decision to postpone their help request for acute health complaints.

## Methods

### Study design and setting

We performed a retrospective observational study consisting of an online questionnaire and telephone interviews with visitors of the ED in the Radboud University Medical Center (Radboudumc), a Dutch level 1 trauma centre with an annual census of 22,000 ED visits in the middle-east of the Netherlands. This study is reported in accordance with the STrengthening the Reporting of OBservational studies in Epidemiology (STROBE) guideline. The local ethics committee ‘CMO region Arnhem-Nijmegen’ approved this study (registration number: 2020–6463). Deidentified datasets are available from the corresponding author upon reasonable requests.

### Data collection

#### Study sample and sampling

Patients who visited the ED between March 9 and July 9, 2020 were retrospectively approached for participation as these dates respectively marked the beginning of the hospital’s registration of patients suspected of or diagnosed with COVID-19, and the ending of the first wave of infections in the Netherlands [15]. All adult patients (≥ 18 years of age) attending the ED for emergency medical care during the predefined inclusion period were eligible for inclusion, except if they were; 1) cognitively impaired or incompetent to participate in a survey, 2) sustained two or more injuries subsequent to a trauma and were transported to the ED via ambulance or trauma helicopter, 3) at an end-of-life stage receiving palliative care, 4) not speaking the Dutch language, or 4) victims of sexual violence. If a patient had more than one ED visit during our study period, this patient was considered a new case and thus included for further data gathering if the initial presenting symptom was new and independent from the previous ED visit(s).

A record of each ED visit during the predefined inclusion period was automatically extracted from the hospital’s electronic patient system and stored in a secured Microsoft Excel database which was only accessible by members of the research team. Pseudonymised basic information from each visit (medical file number, date of birth, date and time of ED arrival, initial ED presenting symptom, corresponding medical specialty, type of ED referral, ED discharge destination and date) were investigated to determine patient’s study eligibility. If cases were deemed eligible, the following additional patient data were gathered; suspected of or diagnosed with COVID-19 (i.e. as registered in the medical chart), visited the ED for a trauma, name and email address. Clinical suspicion of COVID-19 was determined in the ED by physicians assessing the presence of typical and atypical symptoms (i.e., fever, cough, sore throat, diarrhoea, headache, muscle or joint pain, and loss of sense of smell and taste) and based on other signs such as oxygen saturation and lung auscultation findings. Computed tomography (CT) scanning of the thorax and chest radiography (X-ray) were used as aids in the diagnosis of COVID-19. For patients who were hospitalized from the ED the diagnosis of COVID-19 was determined with a reverse transcriptase-polymerase chain reaction (RT-PCR) test. Patients meeting the inclusion criteria were approached within 1 week via email and invited to participate in the survey. This email contained general information about our study, how their personal data is stored and protected, and a link to the online questionnaire. One reminder was sent 5 to 8 days after the initial invitation. Informed consent was implied by completion and return of the questionnaire.

#### Questionnaire

A questionnaire was specifically designed for this study. Multiple draft versions were tested on face-validity and feasibility by three medical students (MN, RvV, IS), two adult patients with a chronic illness, one geriatrician and former head of the ED (YS), and one health scientist (GH), resulting in a final version of the questionnaire (see Additional file 1). The questionnaire consisted of a set of open- and closed-ended questions about: 1) patient’s sociodemographic and clinical characteristics, 2) delay in seeking emergency healthcare; and 3) subsequent motivations for this delay. Data were collected using Limesurvey – a frequently used and secured online questionnaire program – and subsequently transferred to a secured database in the protected server of the Radboudumc.

#### Telephone interviews

RvV and IS conducted short telephone interviews with a sub-sample of respondents who reported delayed healthcare seeking behaviour in the questionnaire and consented with participating in an interview. Interviews were held to better understand respondents’ underlying motivations causing the delay. Respondents were interviewed if their motivations – as described in the questionnaire – were unclear or multi-interpretable. After receiving verbal informed consent for recording the conversation on audio, each interview started with a short recap of the respondent’s questionnaire scores and narratives. Respondents were then asked: “Could you explain what caused the delay in seeking acute medical care for your complaint”? Probes were used if necessary. The interviews were held within 1 week after respondents completed the questionnaire and lasted between 2 to 10 min.

### Data analysis

Descriptive statistics were used to summarize respondent’s sociodemographic and clinical characteristics. Means, standard deviations (SD), medians and Inter Quartile Range (IQR) scores were used for continuous variables. Frequencies and percentages were used for categorical and dichotomous variables. Respondents were compared with non-respondents on: age, gender, ED arrival method, primary medical specialty involved in the ED and COVID-19 suspicion/diagnosis. We used the Pearson’s Chi-squared test for the comparison of categorical and dichotomous data, and the unpaired T-test for continuous data after determining the normality of the data. Statistical significance was set at *p* < 0.05.

Motivations for delayed emergency healthcare seeking behaviour described in the questionnaire were thematically analysed in Microsoft Excel using inductive and deductive reasoning. First, two researchers (MN and GH) independently read all motivations and inductively identified overarching (sub)themes. Irrelevant descriptions such as “I don’t know” or “None” were removed from the data set. The final set of (sub)themes was established after discussion between both researchers. Subsequently, each motivation was deductively categorised into one of the identified subthemes, which resulted in an overview of described motivations organized per subtheme. Relevant interview audio fragments were identified, transcribed verbatim and then categorized under the identified (sub)themes. Fragments were used to illustrate respondent’s motivations.

## Results

### Study sample

Of the 5686 registered ED visits, 4371 cases were identified as eligible for inclusion. Four thousand twenty-five patients finally received the online questionnaire via email and 1338 completed questionnaires were returned, resulting in a response rate of 34.0%. Additional file 2 illustrates the study selection process. Respondents were significantly older than non-respondents (62 versus 57 years, *p* < 0.01; Table 1). Both groups were also significantly different in type of referral to the ED (*p* < 0.01). Groups were fairly similar in gender composition, arriving in the ED during peak hours and being diagnosed with or suspected of COVID-19 in the ED.

**Table 1:** Characteristics of survey respondents and non-respondents among ED visitors during the first wave of the COVID-19 pandemic

Characteristics	Respondents (*n* = 1338)	Non-respondents (*n* = 2687)	*p*-value
Age in years, mean (SD)	61.6 (14.3)	57.2 (18.6)	< 0.01
Gender, n (%)			0.24
*Male*	719 (53.7)	1391 (51.8)	
*Female*	618 (46.3)	1296 (48.2)	
COVID-19^a^, n (%)	336 (25.1)	624 (23.2)	0.19
ED arrival during peak hours^b^, n (%)	644 (48.1)	1293 (48.1)	1.00
Type of referral to the ED^c^, n (%)			< 0.01
*Medical specialist*	478 (35.9)	907 (33.9)	
*GP (transported by ambulance)*	430 (32.3)	774 (28.9)	
*Ambulance*	266 (20.0)	583 (21.8)	
*Self-referral*	73 (5.5)	191 (7.1)	
*Outpatient clinic*	56 (4.2)	104 (3.9)	
*Other hospital or healthcare facility*	21 (1.6)	106 (4.0)	
*Miscellaneous*^d^	6 (0.5)	9 (0.3)	

*ED* emergency department, *GP* general practitioner

^a^Diagnosed with or suspected of COVID-19

^b^ Between noon and 6 pm

^c^ 8 missings in the respondents group and 13 missings in the non-respondents group

^d^ Reported as ‘Other’ in the medical chart

### Respondents with delayed emergency healthcare seeking behaviour

#### Demographic and clinical characteristics

Of the 1338 respondents, 278 (20.8%) reported delayed emergency healthcare seeking behaviour.

(Table 2). Respondents in this group had an average age of 61.6 years (± 13.3 years), most were females (54.7%). More than half (51.1%) arrived during ED peak hours (between noon and 6 pm). Most respondents in this group were referred to the ED by a GP (35.3%) or by a medical specialist (34.5%), and were primarily seen by a cardiologist (40.6%). One in five were suspected of or diagnosed with COVID-19 in the ED.

**Table 2:** Characteristics of ED visitors who delayed in healthcare seeking during the first wave of the COVID-19 pandemic

Characteristics	Respondents (*n* = 278)
Age in years, mean (SD)	61.6 (13.3)
Gender, n (%)	
*Male*	126 (45.3)
*Female*	152 (54.7)
COVID-19^a^, n (%)	70 (25.2)
ED arrival during peak hours^b^, n (%)	142 (51.1)
Type of referral to the ED, n (%)	
*GP (transported by ambulance)*	98 (35.3)
*Medical specialist*	96 (34.5)
*Ambulance*	59 (21.3)
*Outpatient clinic*	12 (4.3)
*Self-referral*	8 (2.9)
*Other hospital or healthcare facility*	4 (1.4)
*Not reported*	1 (0.4)
Corresponding medical specialty, n (%)	
*Cardiology*	113 (40.6)
*Internal medicine*	37 (13.3)
*Pulmonology*	26 (9.4)
*Surgery*	22 (7.9)
*Gastroenterology*	16 (5.8)
*Oncology*	15 (5.4)
*Neurology*	14 (5.0)
*Urology*	12 (4.3)
*Miscellaneous*^d^	23 (10.1)

*ED* emergency department, *GP* general practitioner

^a^Diagnosed with or suspected of COVID-19

^b^ Between noon and 6 pm

^c^≤10 of the cases in each group (i.e., anaesthesiology, geriatrics, haematology, dermatology, gynaecology, intensive care, nephrology, orthopaedic surgery, pain treatment centre, paediatrics, psychiatrics and rheumatology)

### Proportion over time

**Fig. 1:**
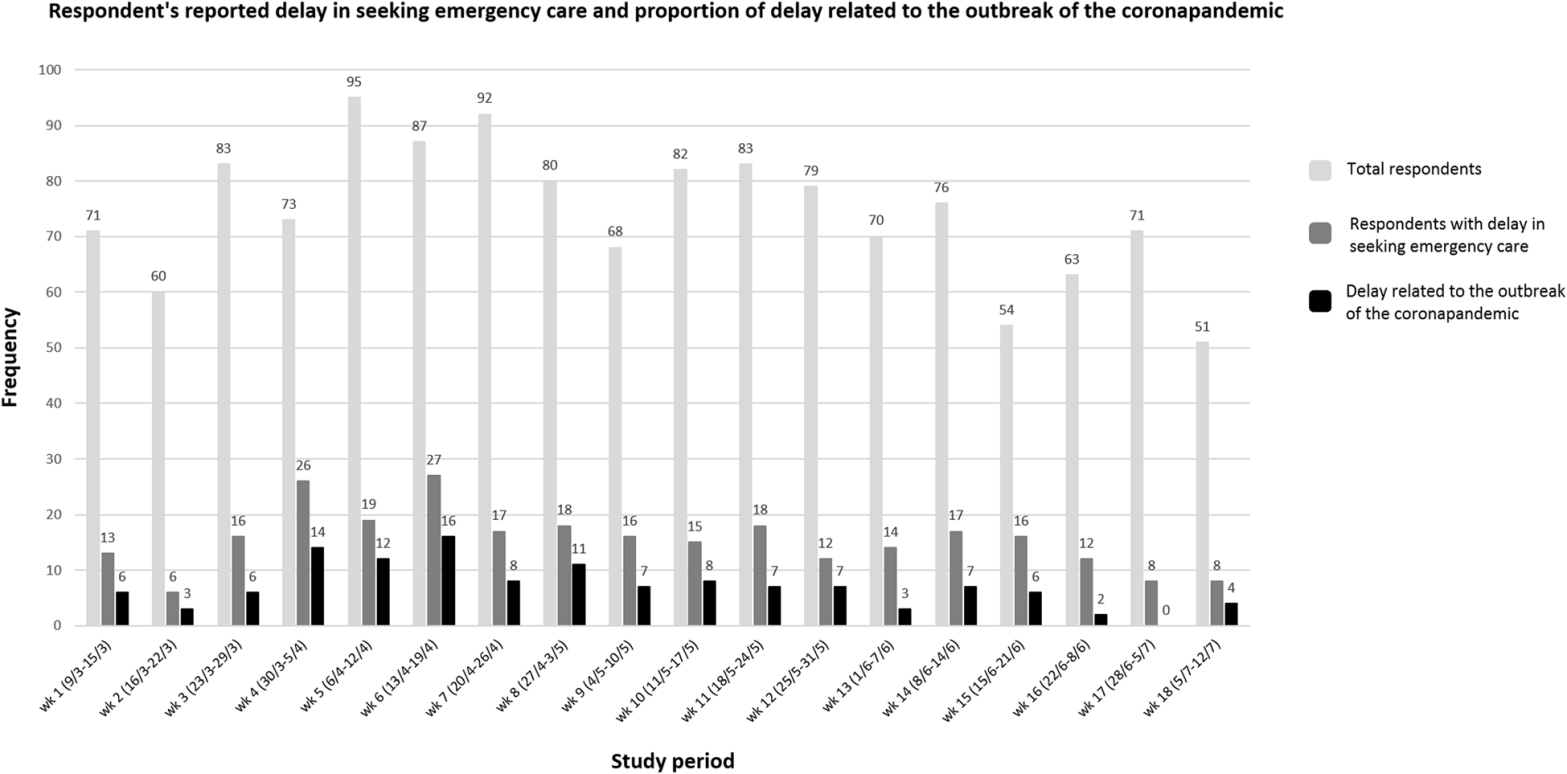
Proportion of respondents with delay in seeking emergency care and delay related to the COVID-19 pandemic

The proportion of respondents reporting delayed emergency healthcare seeking behaviour was relatively consistent throughout the study period, with a peak in week four and six following the COVID-19 outbreak in the Netherlands (Fig. 1). Almost half (*n* = 126; 45.4%) reported that the delay was influenced by the outbreak of the pandemic; this proportion remained fairly consistent throughout the first 15 weeks of the study period.

### Delay and health consequences

The estimated median time of experiencing health complaints before visiting the ED in this group was 5 days (IQR 2–14)*.* The estimated median time of delay in receiving ED care was 3 days (IQR 2–10 days). Within this group, respondents diagnosed with or suspected of COVID-19 and non-COVID-19 respondents did not differ in days of experiencing health complaints before visiting the ED (p = 0.17), and delay in receiving ED care (p = 0.26)*.* Respectively 46 (16.5%) and 26 (9.4%) respondents reported that they believed their health complaints would be either less severe or preventable if they had sought for emergency healthcare at an earlier stage.

### Respondent’s motivations for delayed care seeking behaviour

Two hundred thirty-six respondents described meaningful motivations for their delayed care seeking behaviour. We identified COVID-19 related (*n* = 80; 33.9%) and non-COVID-19 related (*n* = 156; 66.1%) motivations, which were divided into nine subthemes (Table 3). Nineteen respondents were interviewed; their characteristics are summarized in Additional file 3.

**Table 3:** Motivations for delayed healthcare seeking behaviour described by respondents (*n* = 236)

Themes	Subthemes	N (%)^**a**^	Quotes^**b**^
Motivations related to COVID-19	Fear of contamination	36 (15.2)	*“I feared the risk of being contaminated with the virus in the emergency department”. I’m 79, so I won’t take any risk.*
			*“Because I could get in contact with patients having COVID-19 in the waiting room”.*
	Not wanting to put pressure on services	27 (11.4)	*“I knew that the emergency department was overcrowded”.*
			*“I didn’t want to bother the [emergency department] staff with, again, another pancreatitis”.*
	Perceiving own complaints as less urgent/important	6 (2.5)	*“We are dealing with a pandemic! My complaint seemed less urgent and not life-threatening”.*
	Limited access to emergency services	6 (2.5)	*“The outpatient clinic was closed due to the lock-down”.*
			*“I had the impression that the GP was difficult to reach”.*
	Stay home instructions from health professionals	5 (2.1)	*“Because I already had a telephone consult. And I was told that I couldn’t come to the [GP] practice because of corona”.*
Motivations not related to COVID-19	‘Wait-and-see attitude’	114 (48.3)	*“Normally it would go over in a couple of days”.*
	Personal/practical considerations	18 (7.6)	*“Discouraged by previous unsuccessful [hospital]treatments”.*
			*“It’s a 50-km drive for me”.*
			*“I was too sick to realize that I needed acute care immediately”.*
	Following medical advice or treatment plan	17 (7.2)	*“I was told that this would go over on its own”.*
	Complaint develops during out-of-hours	7 (3.0)	*“Because the weekend started. And then the complaints worsened”.*

*GP* General practitioner

^a^Numbers based on motivations described in the questionnaire

^b^From interviews and the questionnaire

A ‘wait-and-see’ attitude (*n* = 114; 48.3%) was most frequently described by respondents. Based on similar experiences in the past and having faith in a quick recovery, they hoped that complaints would subside over time. Delayed care seeking behaviour was also motivated by personal and practical considerations (i.e., too sick, negative experiences with services in the past, travel distance to services), following the medical advice of health professionals or a treatment plan, and the development of complaints during out-of-hours.

Most motivations for delay related to COVID-19 were characterized by the fear of being contaminated with the virus or by the fear of contaminating others. Especially aged and chronically ill respondents expressed this fear as the major cause for waiting longer before seeking acute help. Fuelled by news from traditional and social media, many respondents also described moral concerns as reasons for delay. Respondents did not want to unnecessarily burden emergency care professionals for non-COVID-19 related health complaints. Moreover, they considered their own health complaints as not urgent enough relative to COVID-19 patients. Several respondents mentioned the limited access to services, such as the GP practice and the outpatient clinic, and stay at home instructions from health professionals (because of COVID-19) as major reasons for delay.

## Discussion

The aim of this study was to identify the magnitude, characteristics and underlying motivations for patients’ delayed emergency healthcare seeking behaviour during the first wave of the COVID-19 pandemic in the Netherlands. We found that a relatively large proportion of ED visitors (one in five) reported delay in seeking emergency care during the first wave. For almost half of these visitors the delay was influenced by the outbreak of the pandemic. Numbers of respondents reporting delayed emergency healthcare seeking behaviour were consistently observed throughout the first wave period. These findings are in line with previous studies reporting a substantial drop in ED attendees and delayed ED presentations during the first wave [2, 3, 8, 16], and suggest that since the COVID-19 outbreak many patients with non-COVID-19 related health complaints are less likely to seek emergency healthcare in time, if they even visit the ED at all. Our findings also support the increased concern that new infection waves in the coming months will lead to increased morbidity and mortality risk for those not infected with COVID-19 but in need of ‘regular’ acute care [3, 9]. Interestingly, almost half of these visitors were seen by a cardiologist, suggesting that especially patients with acute heart conditions face the risk of cardiac collateral health damage as a result of delayed care.

To our knowledge, this is one of the first studies to investigate patients’ underlying motivations for delayed emergency healthcare seeking behaviour during the COVID-19 pandemic. The findings show that reasons for delayed care seeking behaviour can be found at the patient, care provider *and* organizational level. Beside the fear of contamination, which has already been addressed in previous studies [6, 8, 17–19], delay is also often caused by patients’ misperceptions of the accessibility of and legitimacy for seeking emergency care in times of a pandemic. Previous studies showed that a patient’s decision to delay coming to the ED often reflects a belief that his or her illness is either self-limited or not serious enough [20, 21]. Comparing own health complaints with COVID-19 severity seems to strengthen these believes. Interestingly, a substantial number of ED visitors experienced delay because they followed the “wait and see” or “stay at home” instructions of their GP or medical specialist. In some cases, these instructions were directly related to COVID-19. This suggests that delayed emergency care also originates from physicians who were reluctant in referring patients to the ED apart from a medical perspective. For example, because they were misinformed about the actual capacity and referral criteria of ED’s in the region.

### Limitations

Our findings need to be interpreted in the light of several limitations. First, this study was performed with visitors from a single ED in the Netherlands. Findings may therefore not be representative for populations at the (inter)national level. Second, this study was confined to one study period. Therefore, the estimated proportion of patients with delayed seeking healthcare behaviour cannot be compared with proportions before the pandemic. Third, the estimated proportion of patients with delayed healthcare seeking behaviour may be biased by selective non-response. Our response rate was rather low and significant differences were found between respondents and non-respondents regarding age and type of referral. The actual proportion of patients with delayed healthcare seeking behaviour may therefore be over- or underestimated. Unfortunately, web-based survey research shows substantial variation in reported response rates and scores below 20% are not uncommon [22, 23]. Nonetheless, we identified a considerable number of ED visitors who experienced delay and described meaningful motivations for their delay. Fourth, data was collected retrospectively and gathered through self-reporting. This may have led to recall bias and a less accurate estimation of delay and perceived health consequences. Fifth, due to practical and financial reasons only patient perceptions were investigated. The perceptions of involved healthcare professionals and relatives could have increased the accuracy and comprehensiveness of findings on motivations for delayed care seeking behaviour and perceived health consequences of the delay. Sixth, sampling bias may have occurred because interview data were only gathered for patients who gave consent for an interview and for those who did not provide a clear description of motivation(s) for delay in the questionnaire. As a result, we may have missed additional insights in motivations for delay that could only be retrieved by performing interviews. Finally, this study is only informative on the magnitude, characteristics and underlying motivations for delayed emergency healthcare seeking behaviour for patients who eventually visited the ED. During the COVID-19 pandemic, there may be a large group of patients with acute health problems that never visited the ED. For example, the number of visits to our ED during the first COVID-19 wave was much lower than normally observed (i.e., a 22% reduction of visits during the study period compared to the same period 1 year ago). Therefore, our results may only show a tip of the iceberg of delayed and missed treatments of acute health problems during the COVID-19 pandemic. This requires attention in future studies.

### Practical implications

Our results indicate the need for proactive strategies to avoid potential avoidance of emergency care services and postponement of help requests by patients with acute health complaints during new infection waves. Strategies should include public outreach in accessible formats tailored for diverse audiences and clearly highlighting the risks of delaying needed care and the established safety precautions in the ED and by other emergency services [21]. Messages should also focus on reassuring the population that emergency care for ‘regular’ acute conditions such as stroke, myocardial infarction and fractures is guaranteed [9, 21]. This can be realized by sparing adequate resources for non-COVID-19 patients and establishing separate care pathways and facilities for COVID-19 and non-COVID-19 patients [17, 21, 24]. Moreover, frequent contact between ED professionals and those responsible for referring or transporting patients to the ED regarding actual ED capacity and referral guidelines could facilitate the timely referral of patients.

## Conclusions

Results from this Dutch single-centre study show that, since the start of the COVID-19 outbreak a relatively large and consistent proportion of ED visitors reported delayed emergency healthcare seeking behaviour. Many of these delayed cases are motivated by patients’ fear for COVID-19 contamination in the ED, and misperceptions of the accessibility of and legitimacy for seeking emergency healthcare in times of a virus outbreak. Health complaints might have been less severe or even preventable if patients would have been seen in the ED at an earlier stage. Public messaging about the risks of delaying needed care, established safety precautions and the low contamination risk in the ED, and clear guidelines for healthcare professionals responsible for ED referral could help to reduce delayed emergency care for patients with acute health problems during future COVID-19 infection waves.

## Supplementary Information

**Additional file 1.** Paper-based copy of the online questionnaire (English version).

**Additional file 2.** Flow-chart of the study selection process

**Additional file 3.** Characteristics of the interviewed respondents

## Data Availability

The datasets generated and analysed during the current study are not publicly available due to anonymity. Deidentified datasets are available from the corresponding author on reasonable request.

## Abbreviations

COVID-19: Coronavirus Disease 2019
ICU: Intensive Care Unit
GP: General Practitioner
ACS: Acute Coronary Syndrome
ED: Emergency Department
STROBE: STrengthening the Reporting of OBservational studies in Epidemiology
SD: Standard Deviation
IQR: Interquartile Range
