# Supplementary material for: Delayed emergency healthcare seeking behaviour by Dutch emergency department visitors during the first COVID-19 wave: a mixed methods retrospective observational study
Source: BMC Emerg Med. 2021 May 1;21:56. doi: 10.1186/s12873-021-00449-9 (PMC8087882; doi:10.1186/s12873-021-00449-9)
Supplement: Supplementary file 1 — Additional file 1. Paper-based copy of the online questionnaire (English version). [file 12873_2021_449_MOESM1_ESM.pdf]

Additional file 1. Paper-based copy of the online questionnaire (translated from Dutch to English)

Thank you for participating in our study. Completing the questionnaire will take approximately 10 minutes of your time.

1. **What is your first (given) and last (family) name?**

   ____

2. **What is your gender?**
   - o Male
   - o Female

3. **What is your age?**

   _______ years

4. **For what health complaint(s) did you seek emergency care?**

   ____

5. **How many days did you experience these health complaint(s) *before* you visited the Emergency Department?**

   _______ days

6. **Did you wait longer to seek for medical help than you would normally do?**
   - o Yes
   - o No (You can skip the next questions)

7. **How many days did you wait longer?**

   _______ days

8. **Why did you wait longer?**

____

9. **Was the delay in seeking for medical help influenced by the outbreak of the COVID-19 pandemic or by news coverage about the coronavirus?**
   - o Yes
   - o No

10. **Do you think that your health complaint(s) would be <u>less severe</u> if you had sought for emergency care at an earlier stage?**
    - o Yes
    - o No

11. **Could your health complaint(s) be <u>prevented</u> if you had sought for emergency care at an earlier stage?**
    - o Yes
    - o No

12. **Can we contact you by telephone for a short interview to better understand the answers you provided in this questionnaire?**
    - o Yes: my telephone number is: ____________
    - o No

Thank you for participating!
