# Supplementary figures and images for: Delayed emergency healthcare seeking behaviour by Dutch emergency department visitors during the first COVID-19 wave: a mixed methods retrospective observational study
Source: BMC Emerg Med. 2021 May 1;21:56. doi: 10.1186/s12873-021-00449-9 (PMC8087882; doi:10.1186/s12873-021-00449-9)

## Additional file 2. Flow-chart of the study selection process

---

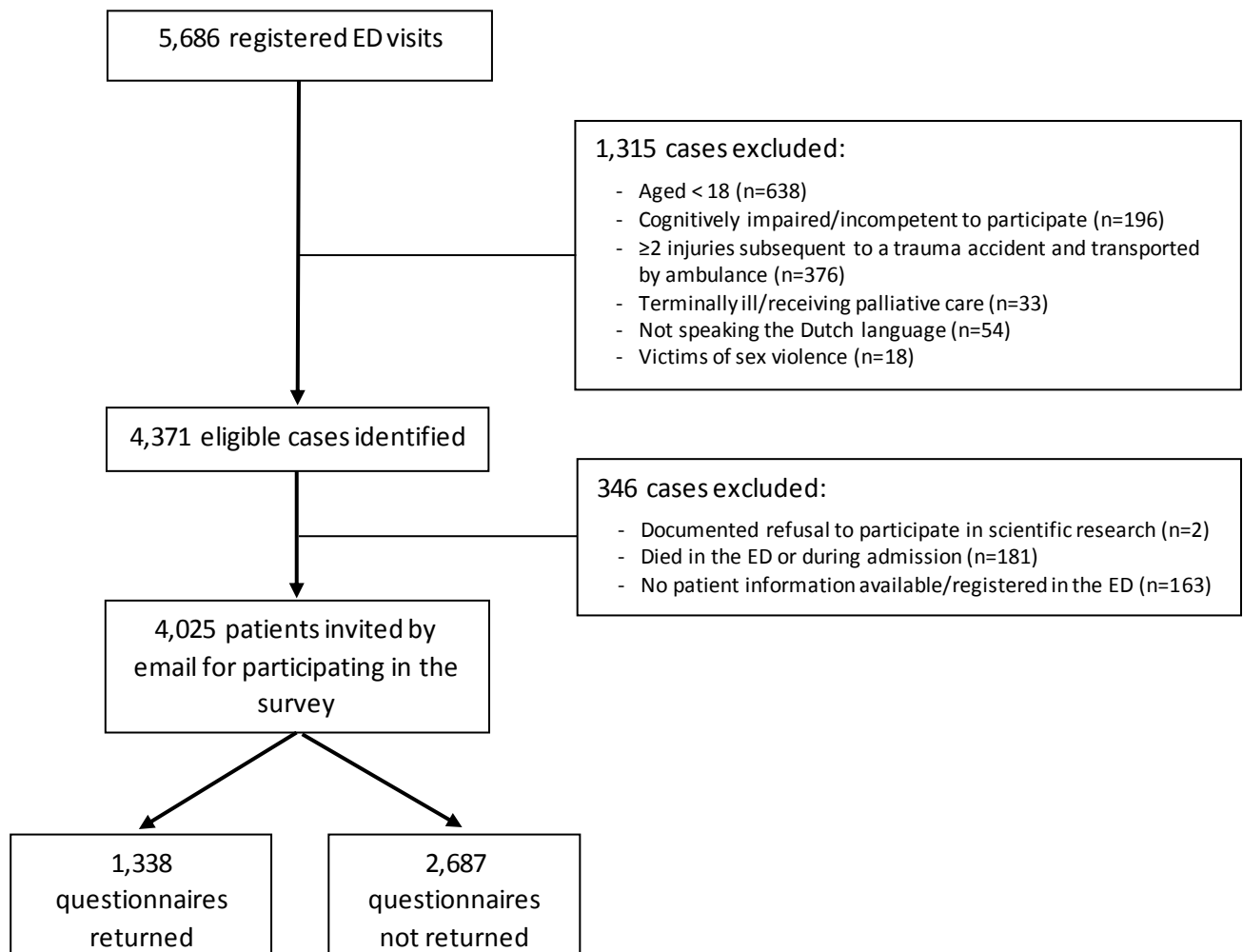

Supplement: Supplementary file 2 — Additional file 2. Flow-chart of the study selection process [file 12873_2021_449_MOESM2_ESM.pdf]
