# Supplementary material for: Delayed emergency healthcare seeking behaviour by Dutch emergency department visitors during the first COVID-19 wave: a mixed methods retrospective observational study
Source: BMC Emerg Med. 2021 May 1;21:56. doi: 10.1186/s12873-021-00449-9 (PMC8087882; doi:10.1186/s12873-021-00449-9)
Supplement: Supplementary file 3 — Additional file 3. Characteristics of the interviewed respondents [file 12873_2021_449_MOESM3_ESM.pdf]

Additional file 3. Characteristics of the interviewed respondents

**Characteristics of the interviewed respondents (n=19)**	
Age in years, median (IQR)	63 (15)
Gender	
Male, n (%)	7 (36,8)
Female, n (%)	12 (63,2)
Medical specialty	
Cardiology, n (%)	11 (57,9)
Pulmonology, n (%)	3 (15,8)
Internal medicine, n (%)	2 (10,5)
Haematology, n (%)	1 (5,3)
Gastroenterology, n (%)	1 (5,3)
Nephrology, *n (%)*	1 (5,3)
Diagnosed with or suspected of COVID-19, n (%)	10 (52,6)
Estimated time of delay in receiving ED care, median (IQR)	6 (11)
Estimated time of experiencing health complaints before visiting the ED, median (IQR)	8 (12)
